# Vaccine Hesitancy Among Religious Groups: Reasons Underlying This Phenomenon and Communication Strategies to Rebuild Trust

**DOI:** 10.3389/fpubh.2022.824560

**Published:** 2022-02-07

**Authors:** Annie Kibongani Volet, Cristina Scavone, Daniel Catalán-Matamoros, Annalisa Capuano

**Affiliations:** ^1^European Programme in Pharmacovigilance and Pharmacoepidemiology, Université de Bordeaux, Bordeaux, France; ^2^Campania Regional Centre for Pharmacovigilance and Pharmacoepidemiology, Naples, Italy; ^3^Department of Experimental Medicine–Section of Pharmacology “L. Donatelli”, University of Campania “Luigi Vanvitelli”, Naples, Italy; ^4^Department of Communication and Media Studies, Madrid University Carlos III, Madrid, Spain

**Keywords:** vaccines, hesitancy, religion, religious reasons, communication strategies

Vaccine hesitancy still represents a phenomenon that undermines the effectiveness of vaccination campaigns and population protection from vaccine-preventable diseases (1, 2). Among reasons underlying this reticence, religion-related convictions probably represent the commonest (3, 4). In this paper we aimed to analyse common religious beliefs connected to vaccine hesitancy and their consequences in terms of vaccination coverage. The need of communication strategies targeted at specific religious populations was analyzed as well. A literature review was carried out in order to achieve study's objectives.

Religious reasons underpinning the vaccine hesitancy were identified for many religious groups, including Protestants, Catholics, Jewish, Muslims, Christians, Amish, Hinduist and Sikhist. For instance, porcine or non-halal ingredients content of vaccines was the main barrier identified in Muslim populations (5–7). Another reason of refusal among Muslims was related to the Ramadan and fasting period. Indeed, during the Ramadan fasting month believers have to abstain themselves from eating, drinking, perfuming or having sexual relationship from sunrise to sunset. A study carried out in Guinea revealed that 46% of Muslims and 80% of religious leaders considered that vaccination was not allowed during the Ramadan. Most cited reasons for refusal were that “Nothing should enter or leave the body during Ramadan” and that “Adverse events could lead to breaking the fast” (8). The belief in a divine fate or to a destiny was found among Muslims. It suggested that someone's disease was the will of God and that nothing should go against it, neither a vaccine (9). Objection to vaccination was also related to: faith in divine protection and healing for Protestants, Catholics, Jewish and Muslims (10); the use of aborted fetal cells for vaccines' production among Amish and Catholic communities (including during the COVID-19 outbreak when Senior Catholic leaders from the US and Canada raised ethical objections to vaccines produced using cells derived from aborted fetuses) (11, 12); the connection between the use of HPV vaccination and sexual promiscuity among Christian parents who consider this vaccine useless for their child as it was considered as a consequence of a certain sexual lifestyle (13, 14). Lastly, the results of the observational, cross-sectional, questionnaire-based study carried out by Sheik A et al. (7) revealed that religious taboos were among the main reasons for non-vaccination among Hinduism and Sikhism believers too.

Vaccine hesitancy driven by religious beliefs brings inevitable consequences for vaccination coverage too. A recent survey carried out in the US that collected HPV vaccination status among American Muslim women (15) showed that 38% of participants received a single dose of HPV while 33% completed the 3-dose schedule. This coverage was below the national estimates of HPV vaccine initiation rates (48–65% as mentioned by the CDC). Conversely, flu shot uptake among American Muslim women was found to be higher than annual adult estimates for a comparable population in the country (71.98% vs. 39–44%). On the other hand, studies analyzing the full immunization status of children showed a higher coverage among religious groups than the rest of the population. Three studies compared religious with non-religious communities in Ghana, Uganda and Zimbabwe in terms of vaccination coverage (16–18). In particular, Budu E et al. reported higher vaccination coverage for children raised in Christian and Muslim families than children from families without religion (16). Similarly, in Uganda, the complete immunization status of children aged 0 to 1-year-old was found to be higher in the Christian community (73.8%) than in the non-Christian one (69.2%) (17). Lastly, the study conducted in Zimbabwe reported the receipt of all basic vaccinations for children aged 12–23 months for the 2010–2011 period of Christians either Apostolic, Roman Catholic, Protestant or Pentecostal/charismatic, Traditionalist and Muslim (18). All those groups had a higher vaccination coverage than participants with no religious affiliation. These considerations emphasize that the individual decision to vaccinate or not among religious groups are not only driven by the religious affiliations since positive trends can be observed among these communities despite known barriers to vaccination.

Notwithstanding these encouraging data on vaccination coverage, we believe that communication strategies targeted at populations specifically concerned are crucial and there is a need for more evaluation of these interventions. Many examples of this type of communication strategies are already in place. For instance, in the scope of the Expanded Programme on Immunization (EPI) in Pakistan, a social mobilization campaign was undertaken to reach community health workers and parents. The objective was to affirm the commitment of the Government in the provision of vaccines and to align the national standards goals and messages toward vaccination. In this campaign, local religious influencers were involved through announcements in Mosque about immunization sessions and through the mentioning of immunization significance during periodic religious sermons (19). A preventive strategy to reduce the incidence of cervical cancer among immunized women in Malaysia consisted in the providing of HPV information followed by a free vaccination. HPV awareness and barriers were assessed through a survey among 13 years old Malaysian girls. The author reported that the overall knowledge regarding HPV vaccine remained poor even after the intervention, since more girls (2.3%) reported that their religion prohibits the HPV vaccine because of its connection with sexual promiscuity (20). Another communication strategy focused on the HPV vaccination was put in place in the US, where the Intermountain West HPV Vaccination Coalition (IWHC) between 10 states and 300 diverse community members was created to improve HPV vaccination among boys and girls and to design new strategies to address HPV barriers, in population of rural and highly religious Intermountain West states (21). Members of the IWHC conducted a survey and focus groups of selected IWHC members about their experience for the 2014–2016 period in the coalition and reported the following top five facilitators to vaccination: strong provider recommendation, improved education about HPV vaccination, increased parental buy-in, focusing on cancer prevention, involving schools more in vaccination.

In conclusion, religious reasons were already known to be sources of vaccine hesitancy. Since vaccination behaviors are not predicted by religion alone but are the results of multiple factors at the individual level, finding the proper effective communication strategy could be a tall order. In order to be effective, we believe that a communication strategy should be based on transparency to build trust, dialogue to involve the targeted community, identify its potential reluctances and address them through scientific exchange of information. The application of the behavior change communication (BCC), as an interactive process aimed to develop tailored messages and promote community behavior change (22), will indeed play a key role in this specific clinical setting.

With these characteristics and together with the continuous monitoring of vaccination coverage, it would be possible to achieve global immunization goals and effectively contrast religious-related vaccine concerns not consistent with scientific knowledge. Lastly, these strategies could contribute to improve vaccination coverage during worldwide emergencies such as the current COVID-19 pandemic.

## Author Contributions

AKV developed the concept. AKV and CS wrote the paper. AKV, CS, DC-M, and AC made substantial contributions to the acquisition, analysis, or interpretation of data for the work and approved the final version of the manuscript to be published. DC-M and AC revised the paper for important intellectual content. All authors contributed to the article and approved the submitted version.

## Funding

This research has received support from the Innovative Medicines Initiative Joint Undertaking under grant agreement n°115014, resources of which are composed of financial contribution from the European Union's Seventh Framework Programme (FP7/2007-2013). More info at https://www.eu2p.org/about-eu2p/project.

## Conflict of Interest

The authors declare that the research was conducted in the absence of any commercial or financial relationships that could be construed as a potential conflict of interest.

## Publisher's Note

All claims expressed in this article are solely those of the authors and do not necessarily represent those of their affiliated organizations, or those of the publisher, the editors and the reviewers. Any product that may be evaluated in this article, or claim that may be made by its manufacturer, is not guaranteed or endorsed by the publisher.
